# Immediate placement of single implant simultaneously with immediate loading in a fresh socket associated to periapical infection: A clinical case report

**DOI:** 10.4317/jced.52160

**Published:** 2015-02-01

**Authors:** Rubén Agustín-Panadero, Blanca Serra-Pastor, Cesar Chust-López, Antonio Fons-Font, Alberto Ferreiroa

**Affiliations:** 1 Associate Professor of the Department of Stomatology. Faculty of Medicine and Dentistry, Valencia University, Spain; 2 Postgraduate student in Prosthodontics. Department of Buccofacial Prostheses. University Complutense of Madrid. Spain; 3 Lab technician. Valencia. Spain; 4 Professor of the Department of Stomatology. Faculty of Medicine and Dentistry, Valencia University, Spain; 5 Associate Professor of the Department of Buccofacial Prostheses. Faculty of Dentistry. University Complutense of Madrid. Spain

## Abstract

Early restoration of the masticatory function, phonatory and aesthetics is some of the current goals of the therapy based on endosseous implants. Facing the classic protocols of implant insertion, which recommend a period of several months between extraction and implant placement, alternatives have been developed that demonstrate that immediate implant placement after tooth extraction permits adequate osseointegration, even in those cases where there is a periapical disease. The immediate restoration of implants after placement is a possibility where aesthetic requirements are high. This article presents a case with immediate implant placement and immediate loading of a first upper premolar with prior periapical pathology due to a vertical fracture. The immediate prosthetic was performed using the extracted crown, which is adapted to be attached to a titanium temporary abutment using a resin cement. After a 4 month healing period work began on the final prosthetic crown. The screw crown was made of zirconium oxide with a covering feldspathic ceramic. At the 12-month follow-up, there were no mechanical or biological complications. The patient gave high satisfaction marks for the overall treatment, giving visual analogue scale score of nine. Immediate post-extraction implants have arisen as an alternative to traditional implants on completely healed bone. Their main aim is to reduce treatment time and number of surgical procedures, along with other objectives such as reduced bone re-absorption and improved aesthetics.

** Key words:**Post-extraction implants, immediate loading prosthetic, implant-retained prosthesis, periapical disease, vertical fracture.

## Introduction

Maxilla alveolar processes are bone structures dependent on the existence of teeth. This bone area will undergo significant structural changes when teeth are lost. The dynamics and magnitude of these changes have been investigated in both animals and in humans. This research has identified the key processes in tissue remodeling after teeth extraction, which can result in a reduction of crest size with significant changes mainly in the buccal bone plate (1).

The biological process that occurs after a tooth extraction produces a physiological reabsorption of the alveolar process and, consequently, a reduction in volume of the maxillary bone, which usually affects the vestibular side of the bone crest. In the first three months following an extraction there will be a horizontal volume reduction of 30% of the alveolar process which could reach up to 50% in 12 months (1,2), hence the need to rebuild oral tissues, is determined by the biological events that occur after teeth extraction.

Immediate post-extraction implants have arisen as an alternative to traditional implants on completely healed bone. Their main aim is to reduce treatment time and number of surgical procedures, along with other objectives such as reduced bone reabsorption and improved aesthetics.

Different authors have proposed different classifications depending on the time elapsed between tooth extraction and implant placement, but all of them agree that the immediate or post-extraction implant is one that is placed in the same surgical procedure the tooth to be replaced is extracted. This concept was introduced by Lazarra (3) 1989 (1989). However, many authors maintain that post-extraction implants are incompatible in cases where the gap between implant and socket is greater than 5 mm (4), as well as in acute and chronic inflammatory periapical processes (5), whereas other authors (6,7), indicate the possibility of implant placement in sockets with periapical inflammatory processes.

## Case Report

**Figure 1:**
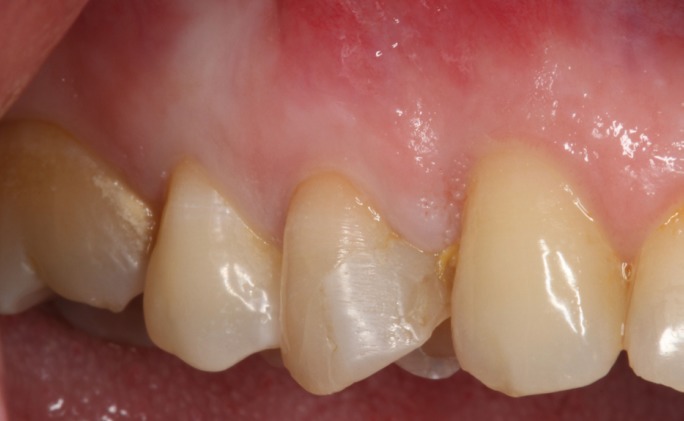
Intraoral view of the tooth 14.

**Figure 2:**
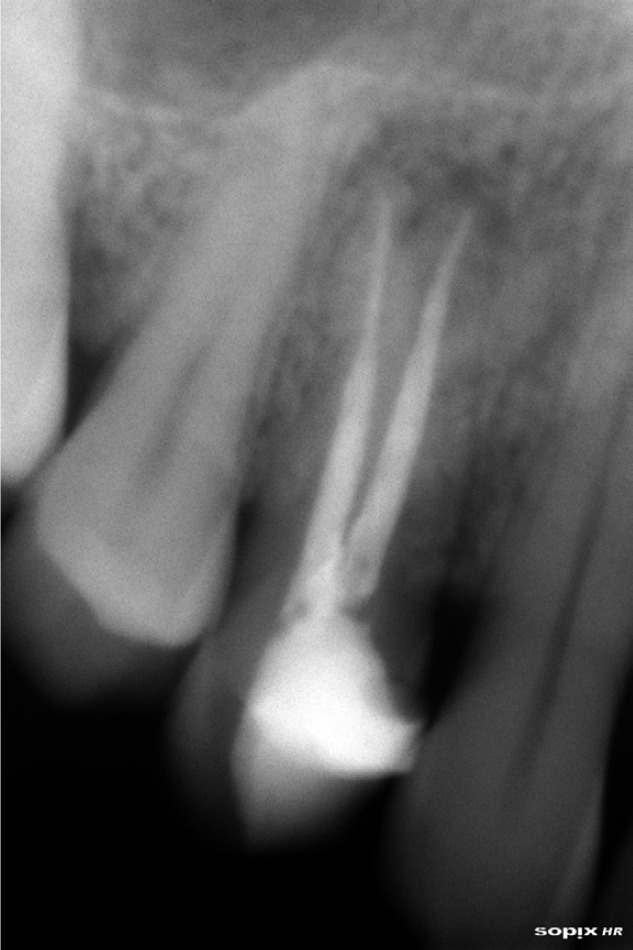
Periapical radiograph show a root canal treatment and periapical lesion.

Female patient, 45, ASA type I, attended our clinic with pain in tooth 14. After clinical examination no abnormalities at gingival level or presence of fistula (Fig. 1) were observed, but percussion pain was present. In the x-ray, root canal treatment with a periapical lesion was observed, making diagnosis compatible with the presence of a vertical fracture and periapical granuloma (Fig. 2). The treatment plan to resolve this case involved the extraction of the tooth (and root canal treatment) with immediate implant placement post extraction and immediate loading to optimize the final restoration esthetics.

**Figure 3:**
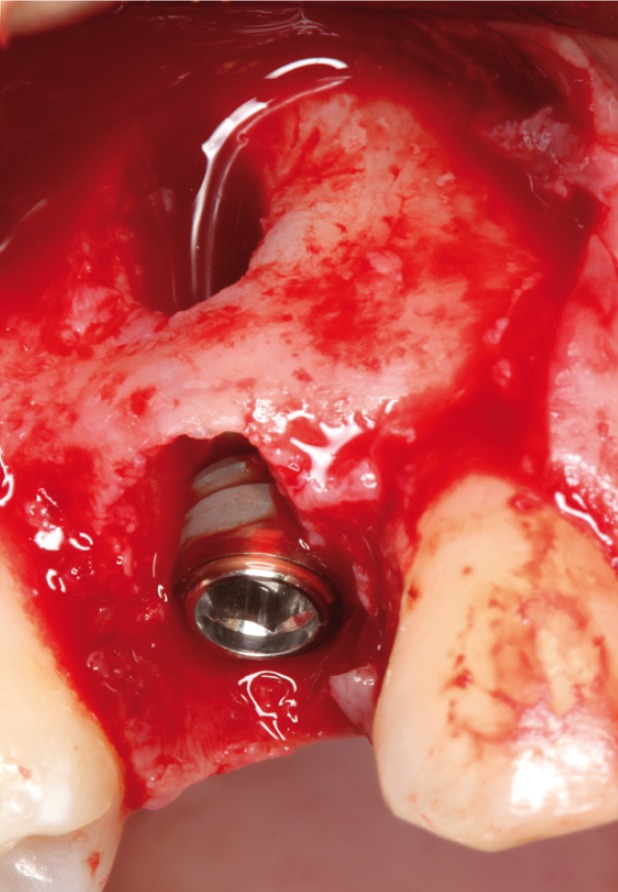
Fenestration in the buccal surface, thet affect more than 50% of the surface of the implant.

**Figure 4:**
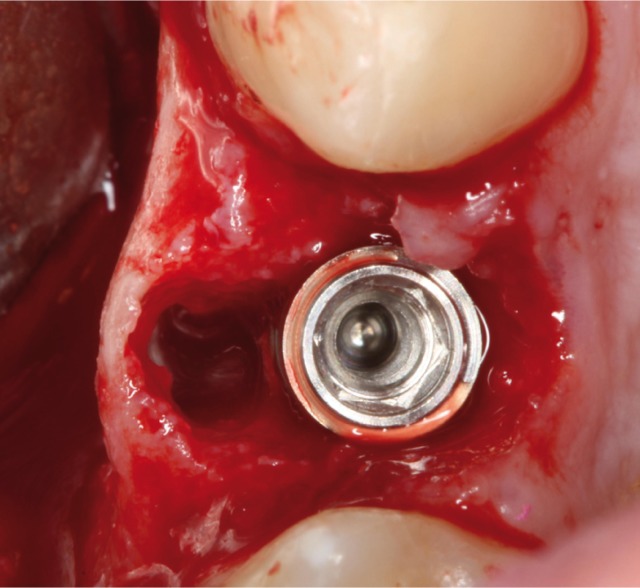
Occlusal view with the gap between implant and buccal face. C. View of the bone graft covering the defect.

Surgery was performed under local anesthesic (4% articaine with 1:100000 adrenaline; Inibsa, Lliça Vall, Catalonia, Spain). After a non-traumatic tooth extraction, the (gum/skin) flap was raised to assess fenestration in the buccal plate and to place a 4,25x13mm implant (Sweden & Martina, Padova, Italy)(Figs. 3,4).

**Figure 5:**
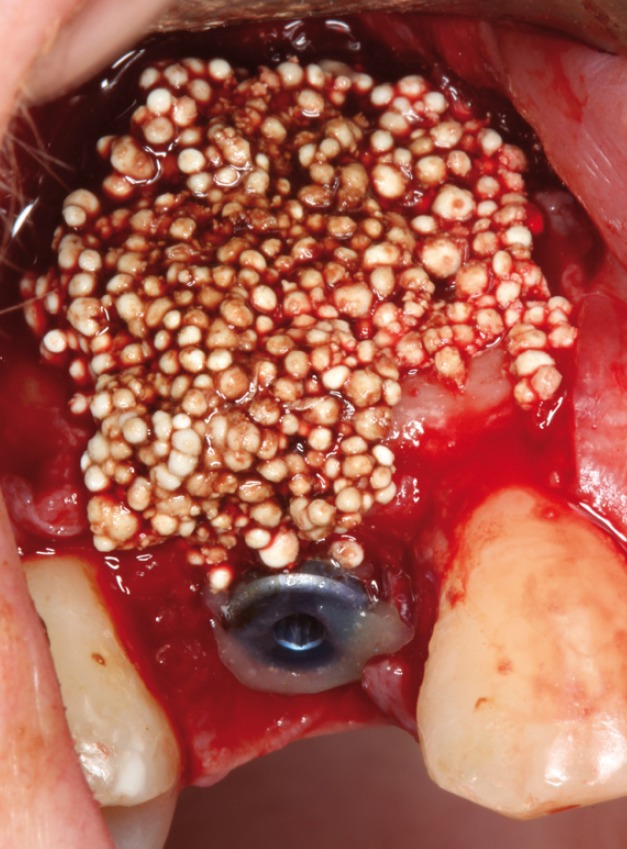
Lateral view of the provisional restoration, using the crown of the extracted tooth.

In the direct observation the apicoronal surface of the implant was only exposed in one wall in a percentage more than 50% (Fig. 5), so that in this case was used a bone graft (Easy-Graft ™CRYSTAL, Sunstar Guidor ®Degradable Solutions AG, Zurich, Switzerland) for covering the fenestration in the buccal face.

The patient was prescribed 1 g amoxicillin (GlaxoSmithKline, Madrid, Spain) twice daily for six days, starting one hour prior to surgery, 600 mg ibuprofen (Bexistar, Laboratorio Bacino, Barcelona, Spain) three times per day for five days and mouth wash with chlorhexidine 0.12% (GUM, John O Butler/Sunstar, Chicago, IL, U.S.A.) twice daily, commencing three days prior to surgery and for two weeks thereafter. Oral hygiene instructions were delivered and a soft diet was recommended for eight weeks. Sutures were removed seven days after the surgery.

-Prosthetic Procedures

**Figure 6:**
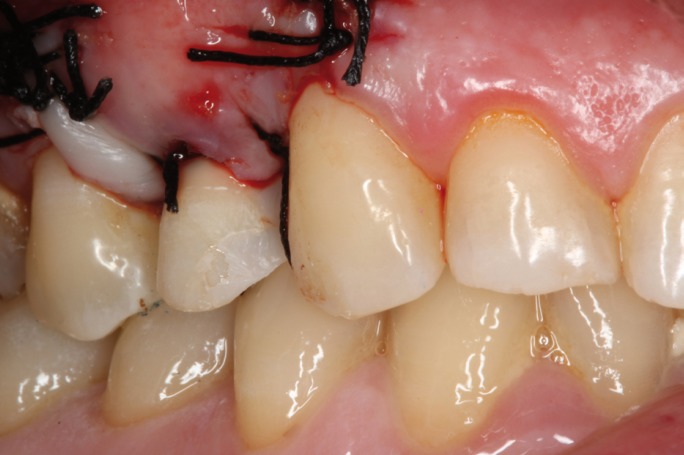
Lateral view of the provisional restoration, using the crown of the extracted tooth.with a success result in the aesthetic of the soft tissues.

**Figure 7:**
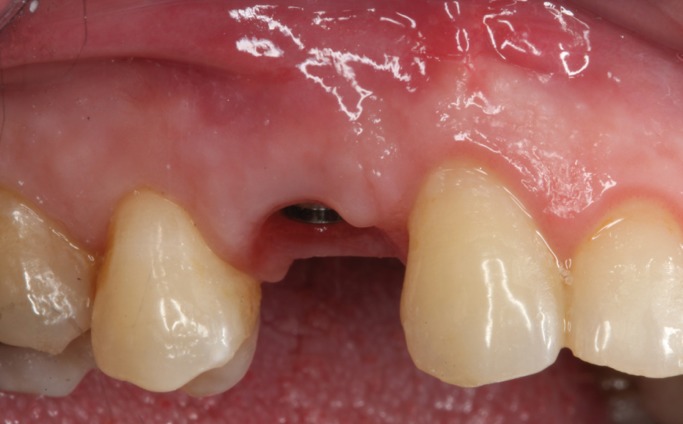
Intraoral view after 4 months of the osseointegration period, with a success result in the aesthetic of the soft tissues.

The immediate temporization was performed using the extracted crown piece, which is adapted to be attached to a titanium temporary abutment using a resin cement (RelyX Unicem cement, 3m ESPE, St Paul MN, USA) (Fig. 6). Previously, the inside of the crown was etched with 37% phosphoric acid. After a 4 month healing period (Fig. 7) work began on the final prosthetic crown. Impressions were taken using the (single-step) double-mix technique with an adittion silicone (Sky and Sky Mix® Heavy Implant Implant Light® silicone fluid (Sweden&Martina®) using the open tray technique.

**Figure 8:**
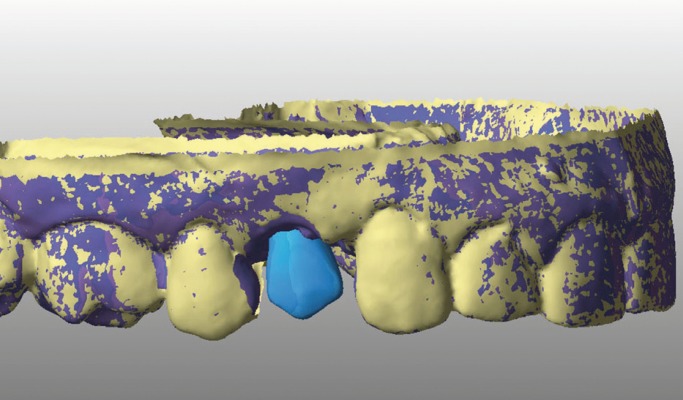
CAD design of the framework of the final restoration.

**Figure 9:**
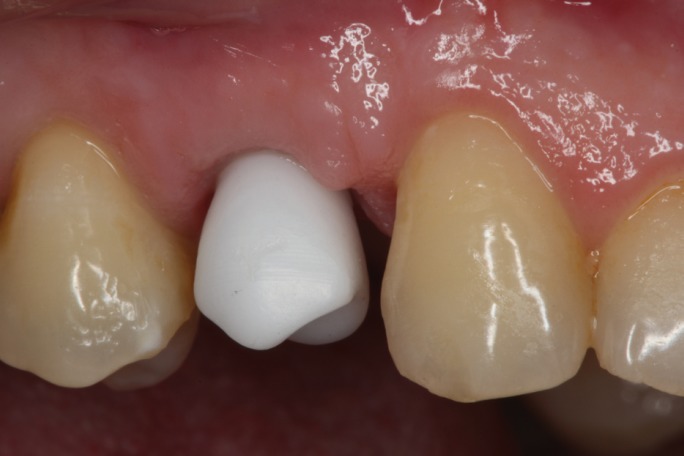
Intraoral view of the screw-retained framework in zirconia.

**Figure 10:**
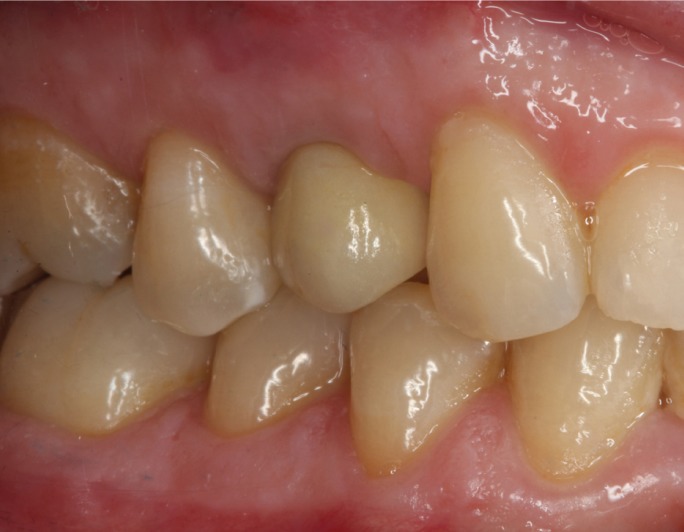
Result of the treatment after 6 months of the placement of the restoration.

Afterwards, the intermaxillary registers and cranio-maxillary transfers were made and mounted on an ARL semi-adjustable articulator Dentatus® set-up (Dentatus USA Ltd., New York, USA) The structure of the screw crown was designed by a CAD design software (Echo Due, Sweden & Martina) (Fig. 8), and made the internal structure out of zirconium oxide (Fig. 9) coated manually with a covering ceramic and cemented by cement resin on a titanium base. On the day of the final placement the occlusion and esthetics were checked (intra-and extra-buccal) and the retaining screw crown was tightened with a pair of 35 N / cm 2 (Fig. 10).

-Follow-up and Patient Satisfaction 

The patient returned for follow-up appointments 1, 6 and 12 months after prosthetic loading. The degree of patient satisfaction was assessed using a 10-cm visual analogue scale (VAS) six months after prosthetic placement. This evaluation assessed general satisfaction with the implant-retained prosthesis, and specific satisfaction regarding comfort, stability, phonetics, ease of cleaning, function, esthetics and self-esteem. The anchor words were “totally dissatisfied” and “completely satisfied.” The patient marked the scale independently, although a research assistant was available to offer help or explanations as needed.

At the 12-month follow-up, there were no mechanical or biological complications. The patient gave high satisfaction marks for the overall treatment, giving VAS score of nine.

## Discussion

The primarily requirement of classic protocol for placing the implants is that the implant site, that is to say, the alveolus, is completely healed after extraction. This technique, apart from the time required for healing after tooth extraction, also needs a healing period after implant placement, making the treatment markedly prolonged in time (1).

This classic technique or protocol for implant placement, has been used since the beginning of the implant placement in order to reduce and minimize the risk of apical bacterial infection, migration and remodeling during early loading (6).

The problem with having long periods of healing time after tooth extraction is the re-absorption that occurs on site. The substantial reduction in bone volume produced in the extraction socket over time can compromise the favorable positioning of the im-plants and their subsequent restoration (8).

To prevent re-absorption in a post-extraction alveolus, Lazzara (3) introduced, for the first time in 1989, a protocol consisting of the placing implants immediately after tooth extraction. This protocol has been widely accepted over time due to the many advantages that it brings; preservation of esthetics, shortening of treatment time, maintenance of alveolar walls, reduction in operating time and the best positioning of the implant (9).

However, using this technique of immediate implant placement after the extraction of a tooth with periapical pathology has been much debated (10,11).

Numerous clinical studies suggest that a socket where a tooth has periodontal or endodontic infection is a marker that predicts infection, and hence the failure of implant treatment. Therefore immediate implant placement is not recommended where there is an infected alveolus (12).

In contrast, numerous studies argue that under controlled conditions, i.e. with certain pre and postoperative measures, immediate implants in infected alveolus can be successful. Most studies that support this method claim that success depends largely on the administration of antibiotics and correct curettage of the alveolus after extraction. Techniques of bone regeneration of defects caused by infection after dental implant placement (9,10,12) are also proposed.

In a study by Lindeboom *et al.* (10) whose purpose was to determine the clinical success of implant placement in alveolus with chronic periapical infection, registered survival values, stability, gingival aesthetics and radiographic bone loss in 2 groups; one of immediate implants in infected extraction alveolus and the other of implants in alveoli where there had previously been infection. Survival values of 92% for immediate implants were obtained and no significant differences were found in terms of stability, gingival aesthetics and radiographic bone loss.

The placement of a temporary prosthesis prior to placement of a definitive prosthesis can allow the tissue to grow faster and take on the definitive gingival form as it can be modified over several appointments to achieve the desired formation (13).

Schwartz-Arad and Chaushu (14,15), in their literature review on immediate implants describe survival rates, for the same groups, of 93.9% to 100%. That same year, the same author (16), in a retrospective study of 7 years of follow-up obtained a success rate of 95%. Subsequently, Chaushu *et al.* (17), in a clinical study comparing immediate versus non-immediate implantation obtained a success rate for the former of 82.4 percent, and for non-immediate implants 100%. Perry *et al.* (18) in a 5-year retrospective evaluation, which compared immediate implants with non- immediate implants obtained survival rates of 90.03 percent and 90.04 percent respectively. This technique is supported by literature with high survival rates reported by Becker *et al.* (19) (97.2% percent), Wagenberg and Froum (20) (96% percent).
